# Neonatal sepsis: a systematic review of core outcomes from randomised clinical trials

**DOI:** 10.1038/s41390-021-01883-y

**Published:** 2022-01-07

**Authors:** Cían J. Henry, Gergana Semova, Ellen Barnes, Isabel Cotter, Tara Devers, Aisyah Rafaee, Andreea Slavescu, Niamh O. Cathain, Danielle McCollum, Edna Roche, David Mockler, John Allen, Judith Meehan, Claus Klingenberg, Jos M. Latour, Agnes van den Hoogen, Tobias Strunk, Eric Giannoni, Luregn J. Schlapbach, Marina Degtyareva, Frans B. Plötz, Willem P. de Boode, Lars Naver, James L. Wynn, Helmut Küster, Jan Janota, Fleur M. Keij, Irwin K. M. Reiss, Joseph M. Bliss, Richard Polin, Joyce M. Koenig, Mark A. Turner, Christopher Gale, Eleanor J. Molloy, On behalf of the Infection, Inflammation, Immunology and Immunisation (I4) section of the European Society for Paediatric Research (ESPR)

**Affiliations:** 1 Discipline of Paediatrics, Trinity College Dublin, The University of Dublin & Children’s Hospital Ireland (CHI) at Tallaght, Dublin, Ireland; 2 John Stearne Medical Library, Trinity College Dublin, St. James’ Hospital, Dublin, Ireland; 3 Trinity Translational Medicine Institute, St. James Hospital, Dublin, Ireland; 4 Trinity Research in Childhood Centre (TRiCC), Trinity College Dublin, Dublin, Ireland; 5 Department of Pediatrics and Adolescence Medicine, University Hospital of North Norway, Tromsø, Norway; 6 Paediatric Research Group, Faculty of Health Sciences, UiT-The Arctic University of Norway, Tromsø, Norway; 7 School of Nursing and Midwifery, Faculty of Health, University of Plymouth, Plymouth, UK; 8 Division of Woman and Baby, Department of Neonatology, Wilhelmina Children’s Hospital (part of UMC Utrecht) and University of Utrecht, Utrecht, The Netherlands; 9 Neonatal Health and Development, Telethon Kids Institute, Perth, WA Australia; 10 Neonatal Directorate, King Edward Memorial Hospital for Women, Perth, WA Australia; 11 Clinic of Neonatology, Department Mother-Woman-Child, Lausanne University Hospital and University of Lausanne, Lausanne, Switzerland; 12 Paediatric Critical Care Research Group, Child Health Research Centre, University of Queensland, Brisbane, QLD Australia; 13 Paediatric Intensive Care Unit, Queensland Children’s Hospital, Brisbane, QLD Australia; 14 Department of Pediatrics, Bern University Hospital, University of Bern, Bern, Switzerland; 15 Department of Neonatology, Pirogov Russian National Research Medical University, Moscow, Russia; 16 Department of Paediatrics, Tergooi Hospital, Blaricum, The Netherlands; 17 Department of Paediatrics, Amsterdam UMC, Emma Children’s Hospital, University of Amsterdam, Amsterdam, The Netherlands; 18 Department of Neonatology, Radboud Institute for Health Sciences, Radboud University Medical Center, Amalia Children’s Hospital, Nijmegen, The Netherlands; 19 Karolinska University Hospital and Karolinska Institutet, Stockholm, Sweden; 20 Department of Paediatrics, University of Florida, Gainesville, FL USA; 21 Department of Pathology, Immunology, and Laboratory Medicine, University of Florida, Gainesville, FY USA; 22 Department of Neonatology, Clinic for Paediatric Cardiology, Intensive Care and Neonatology, University Medical Centre Göttingen, Göttingen, Germany; 23 Neonatal Unit, Department of Obstetrics and Gynecology, Motol University Hospital and Second Faculty of Medicine, Prague, Czech Republic; 24 Institute of Pathological Physiology, First Faculty of Medicine, Charles University, Prague, Czech Republic; 25 Department of Pediatrics, Division of Neonatology, Erasmus MC-Sophia Children’s Hospital, Rotterdam, The Netherlands; 26 Department of Pediatrics, Women & Infants Hospital of Rhode Island, Alpert Medical School of Brown University, Providence, RI USA; 27 Division of Neonatal-Perinatal Medicine, Department of Pediatrics, Columbia University Medical Center, New York City, NY USA; 28 Division of Neonatology, Edward Doisy Research Center, Saint Louis University, St. Louis, MO USA; 29 Institute of Translational Medicine, University of Liverpool, Centre for Women’s Health Research, Liverpool Women’s Hospital, Liverpool, UK; 30 Department of Neonatal Medicine, School of Public Health, Faculty of Medicine, Imperial College London, Chelsea and Westminster Campus, London, UK; 31 Department of Paediatrics, Coombe Women’s and Infant’s University Hospital, Dublin, Ireland; 32 Department of Neonatology, CHI at Crumlin, Dublin, Ireland

## Abstract

**Background:**

The lack of a consensus definition of neonatal sepsis and a core outcome set (COS) proves a substantial impediment to research that influences policy and practice relevant to key stakeholders, patients and parents.

**Methods:**

A systematic review of the literature was performed according to the Preferred Reporting Items for Systematic Reviews and Meta-Analyses (PRISMA) guidelines. In the included studies, the described outcomes were extracted in accordance with the provisions of the Core Outcome Measures in Effectiveness Trials (COMET) handbook and registered.

**Results:**

Among 884 abstracts identified, 90 randomised controlled trials (RCTs) were included in this review. Only 30 manuscripts explicitly stated the primary and/or secondary outcomes. A total of 88 distinct outcomes were recorded across all 90 studies included. These were then assigned to seven different domains in line with the taxonomy for classification proposed by the COMET initiative. The most frequently reported outcome was survival with 74% (*n* = 67) of the studies reporting an outcome within this domain.

**Conclusions:**

This systematic review constitutes one of the initial phases in the protocol for developing a COS in neonatal sepsis. The paucity of standardised outcome reporting in neonatal sepsis hinders comparison and synthesis of data. The final phase will involve a Delphi Survey to generate a COS in neonatal sepsis by consensus recommendation.

**Impact:**

- This systematic review identified a wide variation of outcomes reported among published RCTs on the management of neonatal sepsis.
- The paucity of standardised outcome reporting hinders comparison and synthesis of data and future meta-analyses with conclusive recommendations on the management of neonatal sepsis are unlikely.
- The final phase will involve a Delphi Survey to determine a COS by consensus recommendation with input from all relevant stakeholders.

## Introduction

Neonatal sepsis is estimated to be responsible for 15% of all neonatal deaths globally.^1^ It is a source of significant morbidity including delayed enteral feeding, prolonged duration of mechanical ventilation and hospital stay and long-term disability, and the sequelae may extend well into childhood and last throughout life.^2^

The selection of appropriate outcomes or endpoints is crucial when designing clinical trials in order to directly compare the effects of different interventions or studies. If the findings are to influence policy and practice, then the chosen outcomes need to be relevant and important to key stakeholders, including patients, parents and the public, healthcare professionals and other decision-makers in healthcare.^3^

A proposed solution to this issue of inconsistent outcome reporting in neonatal sepsis is the development and implementation of a standardised core outcomes set (COS), defined as ‘the minimum that should be measured and reported in all clinical trials of a specific condition’.^4^ COS have been developed in gastroschisis and neonatal nutrition to standardise reporting and the selection of outcomes.^5,6^ The *Core Outcomes in Neonatology* project is developing a COS for neonatal medicine to establish standard measures, which are important to key stakeholders, clinically relevant and reported consistently in future trials.^7^ Furthermore, developing standardised outcome sets for testing interventions is recommended including a general agreement about uniform time points of the measurements.^8^

The lack of an accepted consensus definition and COS for neonatal sepsis are substantial impediments to research to improve diagnosis, outcomes and prognosis. Inconsistent outcome selection and reporting limit the usefulness of clinical trials that cannot be compared or combined in the synthesis of a meta-analysis or systematic review.^9^ The lack of a standardised COS and standard measures limits the ability of meta-analyses to answer clinically meaningful questions and clinical practice lacks high-quality evidence or international evidence-based consensus guidelines.^10^

A proposed solution to this issue of inconsistent outcome reporting in neonatal sepsis is the development and implementation of a standardised COS, defined as ‘the minimum that should be measured and reported in all clinical trials of a specific condition’.^4^ COS have been developed in gastroschisis and neonatal nutrition to standardise reporting and the selection of outcomes.^5,6^ The Core Outcomes in Neonatology project is developing a COS for neonatal medicine to establish standard measures that are important to key stakeholders, clinically relevant and reported consistently in future trials.^7^ Furthermore, developing standardised outcome sets for testing interventions is recommended, including a general agreement about uniform time points of the measurements^8^

The aim of this systematic review was to identify outcomes reported in published randomised controlled trials (RCTs) of therapeutic interventions in neonatal sepsis. This phase of research is part of a wider protocol in the development and implementation of a COS for neonatal sepsis that will standardise the selection, recording and reporting of outcomes, ultimately translating into improved neonatal care.^11,12^

## Materials and methods

We prospectively registered the study on the Core Outcome Measures in Effectiveness Trials (COMET) database for COS. A systematic review was undertaken to identify outcomes that have been reported in RCTs. Ethical approval was not required.

### Literature search

All RCTs investigating the management of suspected or confirmed neonatal sepsis in a hospital inpatient setting since 1982 were included. Studies investigating measures used to prevent neonatal sepsis were not included. A database search of MEDLINE, Embase and Web of Science was undertaken using standardised search terms. This review was conducted in line with Preferred Reporting Items for Systematic Reviews and Meta-Analyses (PRISMA) guidelines.^13^

### Data extraction

Using the Covidence® software, two authors (C.J.H. and E.B.) independently screened the titles, abstracts and full papers regarding the inclusion and exclusion criteria. Studies without data for retrieval or duplicate publications were excluded. Any disputed articles were discussed among the full research group. Outcomes from included trials were extracted verbatim in accordance with the provisions of the COMET handbook.^14^ A structured and pilot-tested data extraction tool, specific to this review, was developed. This tool was used to tabulate primary and secondary outcomes/endpoints, which we extracted and grouped into outcome domains in line with COMET guidelines. The tool also recorded: author, date, number of centres, number of patients, exclusion and inclusion criteria, method of randomisation and blinding, number of treatment arms, intervention under investigation and timeframe.

### Data synthesis

Outcomes were categorised into seven different domains (depending on whether a biological activity or clinical benefit was being recorded) according to the framework of the OMERACT 2.0 filter.^15,16^ Domains represent an aspect of health or a health condition that needs to be measured to appropriately assess the effects of a health intervention. The framework consists of levels such as pathophysiological manifestations and death. An outcome matrix based on the Outcome Reporting Bias in Trials project,^17^ and as recommended by the COMET initiative,^14^ was constructed to visually represent the frequency, consistency and disparity of outcome reporting across studies (Fig. 2).

## Results

**Fig. 1:**
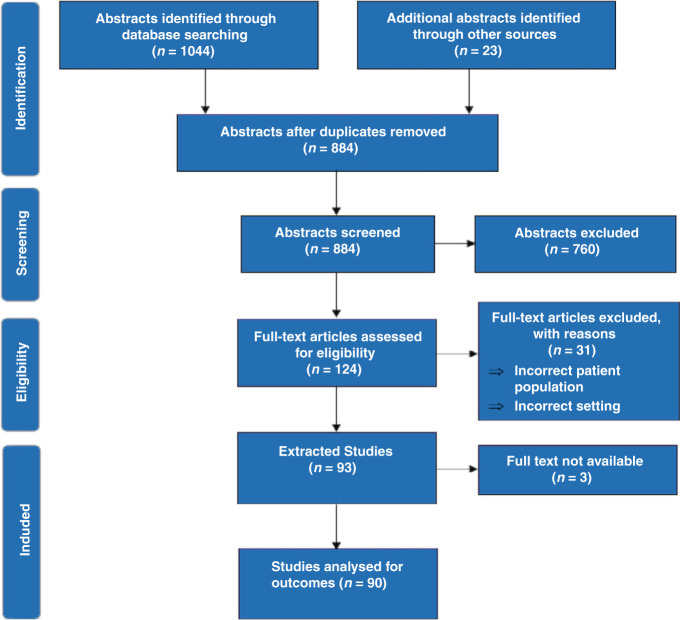
PRISMA flow diagram. Flow diagram detailing systematic selection of the published literature.

The search strategy retrieved 1067 studies. One hundred and eighty-three duplicates were removed yielding 884 papers for screening of title and abstract. One hundred and twenty-four papers were included for full-text screening and a total of 90 RCTs were extracted for detailed analysis (Fig. 1).^18–107^ Thirty papers explicitly stated their primary and/or secondary outcomes. A total of 88 distinct outcomes were recorded among the 90 trials included. Twenty-four distinct types of outcome measures were recorded across 22 out of the 90 included RCTs and only three of these measures could be directly compared with others used in different trials. Eleven of the included trials used composite outcomes and six of these were the primary outcomes of the study.

### Mortality

The most frequently reported outcome was survival in 67/90 (74%) trials. However, amongst these 67 studies, 27 different survival outcomes were used with a varying array of time points from 3 days until 2 years. From the 67 trials reporting mortality, 42 reported all-cause mortality, 7 reported disease-specific mortality (e.g. sepsis) and 23 reported mortality within a certain time period.

### Morbidity

**Fig. 2:**
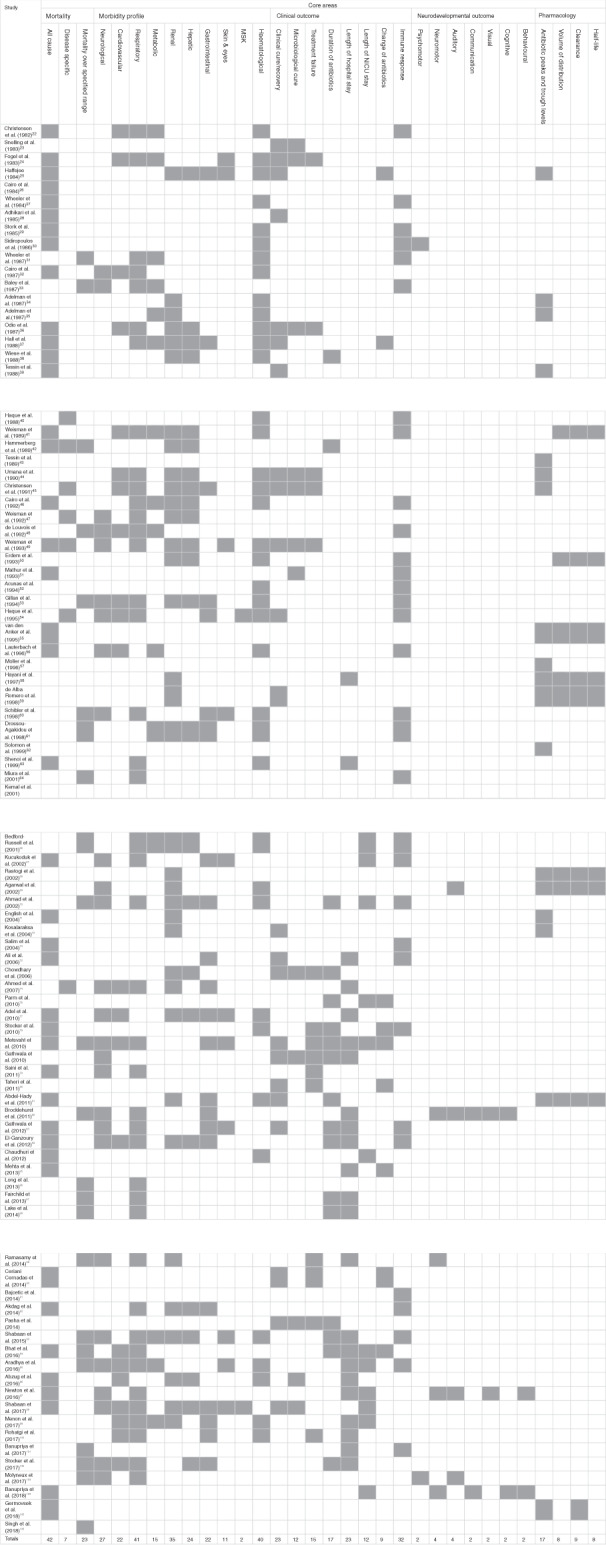
Matrix of outcomes reported by each study of neonatal sepsis. The grey box with shading indicates that this outcome was reported). Only the first author of each study is given. Five core areas (mortality, morbidity, clinical outcome, neurodevelopmental outcome, pharmacology) have been selected to categorise these outcomes as they represent the domains most frequently reported in the included studies. MSK musculoskeletal.

**Fig. 3:**
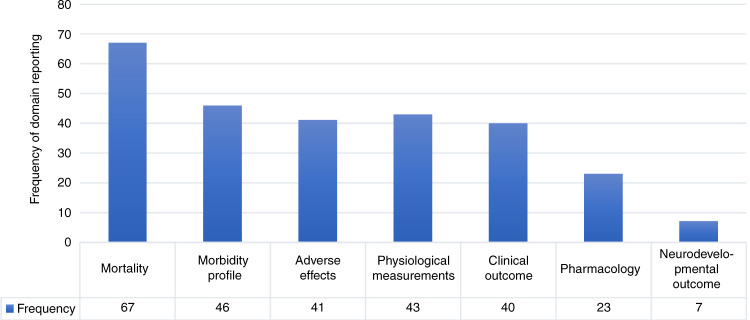
Frequency of domain reporting across the 90 included studies of neonatal sepsis. The number of trials reporting outcomes within each of these seven domains is represented. Mortality 74% (67/90), morbidity profile 51% (46/90), adverse effects 46% (41/90), physiological measurements 48% (43/90), clinical outcome 44% (40/90), pharmacology 25% (23/90) and neurodevelopmental outcome 8% (7/90).

Outcomes related to clinical improvement (44%) and morbidity profiles (51%) were also frequently reported in the included studies (Figs. 2 and 3). The morbidity profile of patients was categorised into organ-specific domains according to the organ function affected. Forty-two of the 90 (46.7%) studies reported morbidity outcomes related to the respiratory system such as pneumonia, duration of mechanical ventilation and chronic lung disease. Another domain was the haematological system with 39 (43.3%) studies reporting outcomes. Thirty-two out of 90 studies investigated the immune response to the treatment of neonates with sepsis, particularly studies investigating granulocyte colony-stimulating factor (CSF) administration where laboratory outcomes such as absolute neutrophil count, time to immune recovery and the ratio of immature-to-total granulocytes were reported.

Gastrointestinal morbidity outcomes such as necrotising enterocolitis and time to enteral feeding were reported in 24 studies and neurological morbidity outcomes such as meningitis and intraventricular haemorrhage in 27 studies. The reporting of outcomes related to hepatic and renal morbidity was frequently seen in studies investigating the efficacy and safety of different antibiotic regimens with 24 and 35 studies, respectively.

### Clinical outcome

Clinical cure or recovery was reported as an outcome in 23 studies, but this was inconsistently measured with no standardisation across studies. A smaller number of studies used microbiological cures (e.g. eradication of organism from blood culture, CSF culture, etc.) with only 11 reporting on this outcome. Duration of hospital stay (*n* = 23) and duration of neonatal intensive care unit stay (*n* = 12) was reported in a number of the included studies and categorised within the clinical outcome domain. Treatment failure (*n* = 15), duration of antibiotic therapy (*n* = 17) and change of antibiotic therapy (*n* = 9) were less commonly reported in the included studies. The definition of treatment failure as an outcome measure, like clinical recovery, varied widely across the included studies with wide heterogeneity and no standardisation. Fifteen out of ninety studies reported treatment failure as an outcome.^34^

Only 7/90 (7.8%) studies reported neurodevelopmental outcomes and again we found heterogeneity in terms of the outcome measures applied across the studies where this was reported. Composite outcomes were used in 11/90 (12.2%) of the studies and the most common was combining clinical and microbiological criteria used to determine clinical response after the treatment of sepsis. There was significant heterogeneity among the composite endpoints recorded, where a number of incomparable composite outcome measures were reported in a single study only. Five of these measured sepsis in a manner that considered organ dysfunction as part of the composite endpoint, but a comparison between all the studies could not be made as three distinct scoring systems were used.

## Discussion

There is a wide variation of outcomes among published RCTs on the management of neonatal sepsis. The paucity of standardised clinical outcomes in neonatal sepsis limits evidence synthesis and systematic review. Therefore, long-term follow-up is needed in trials on neonatal sepsis to provide evidence on how to optimise outcomes throughout childhood and into later life.^108,109^

Mortality is challenging to record as an outcome, as the cause of death in critically ill neonates cannot always be attributed solely to infection as opposed to underlying disease or a combination of both. While mortality is a SMART outcome, mortality rates in preterm neonatal sepsis studies are affected by many other conditions, and causality may be hard to prove. In addition, major morbidities in survivors have been shown to greatly affect long-term outcomes, implying a need for robust non-mortality COS. The majority of studies included in our analysis did not report a time interval between infection and death and survival definitions ranged from 3 days to 2 years. This disparity is undesirable as short-term survivals may fail to include late disease-related deaths, while long-term survivals may be confounded by other causes of death. Most deaths from sepsis in neonates occur within 5 days of disease onset.^110,111^ Furthermore, the use of mortality as the sole or primary endpoint has its own limitations due to the fact that for patients with major life-altering co-morbidities, mortality might not be the most significant patient-centred outcome.^112^ Only a small number of trials reported neurodevelopmental outcomes.

All-cause mortality or death due to infection was used in some studies. If all-cause mortality is used as an endpoint, causes of death unrelated to sepsis may be captured within the study population and could impact the interpretation of the results. To overcome this, composite endpoints including mortality and morbidity parameters have been proposed as a more appropriate primary endpoint. The Bayley Scales of Infant Development at 18 months to 2 years is cited as a gold standard for neurodevelopmental outcomes and potentially a useful endpoint in neonatology trials, but many trials use a composite of death and neurodevelopmental outcome to reduce sample size estimates.^113^ Composite outcomes have been criticised because they can be considered clinically meaningless,^108^ may either artificially inflate effect sizes or mask potentially important effects seen in one individual outcome of the composite.^114^

Other primary outcomes were frequently defined as subjective clinical cure and/or improvement and bacterial eradication with 25 included studies reporting this outcome. The definitions of clinical cure showed vast heterogeneity between each of these 25 studies. Most used a broad overall assessment of clinical findings by the attending neonatologist, with only five papers^34,56,63,80,91^ utilising a standardised clinical assessment tool such as the Score for Neonatal Acute Physiology^115^ and Tollner’s sepsis score (clinical and basic laboratory scoring parameters).^116,117^ The efficacy of measuring these forms of clinical alteration depends on the measure used, e.g. length of ICU stay, which is complicated by differences in criteria for ICU discharge and the availability of non-ICU beds in an institution. Other endpoints included organ dysfunction and morbidity-free days. Morbidity-free days (ventilator, dialysis and other organ dysfunction-free days) require complex statistical models, but may be able to detect clinically important effects.

Forty-three of the ninety included studies utilised laboratory measurements to assess outcome, with inflammatory markers and acute-phase reactants being the most commonly utilised measures. Two of the ninety studies reported on the use of procalcitonin as a biomarker to assess outcome.^74,98^

The reporting of outcomes relating to morbidity and organ dysfunction were poorly characterised throughout the included trials, particularly in relation to neurodevelopmental outcomes. Heterogeneous and incomplete reporting of organ failure in the neonatal critical care setting creates problems when interpreting and applying the findings to practice as organ failure is integral to the pathophysiology of sepsis and core feature of the definition in adults but not well-defined in neonates. This observation may reflect the difficulties inherent to the RCT in detecting long-term outcomes.

The multitude of inconsistent outcomes render the findings of systematic review or meta-analysis on the topic inconclusive,^10^ and unless outcome selection can be re-organised or standardised to reflect consistent and comparable measures, future meta-analyses with conclusive recommendations on the management of neonatal sepsis are unlikely.

Oeser et al. performed a systematic review of clinical trials in neonatal sepsis and identified the subjective clinical cure and/or improvement and bacteriological eradication as most primary reported endpoints. The authors discussed the lack of validated core outcomes and suggested composite endpoint including clinical and laboratory parameters as most appropriate COS for future trials. However, their recommendations have not been universally adopted in subsequent trials.^118,119^ This review differed from our study with narrower inclusion criteria for trials and fewer overall papers analysed. Our systematic review constitutes one of the initial phases in the development of a protocol for a COS in neonatal sepsis. The authors have identified the existing knowledge and outcomes already reported. Given the paucity of standardised outcome reporting in this area, the need for a COS is readily apparent.

While some guidelines emphasise the role of parental concern in recognising sepsis, a paucity of data in the field is evident. Therefore, an improved understanding of whether parental concerns adds diagnostic value to sepsis recognition is urgently needed. Including parental concerns in sepsis screening tools could benefit the assessment resulting in early diagnosis and treatment of infants with sepsis.^120^

Currently, there is still no widely accepted definition of neonatal sepsis. The Third International Consensus Definitions for Sepsis and Septic Shock (Sepsis-3)^121,122^ reflects the complex pathophysiology of sepsis as a life-threatening organ dysfunction caused by a dysregulated host response to infection. This definition has now been applied in children and adults and does not require a microbiological diagnosis.^123^ Neonatal sepsis, however, is most commonly defined based on microbiological detection of a pathogen and/or a combination of clinical signs with or without the addition of biomarkers and is in contrast to the adult and paediatric settings.

The next phase will involve developing a protocol for a COS in neonatal sepsis, resolving gaps in the existing knowledge and assessing the quality of studies and outcomes already reported, including uniform time points of the measurements and standardised parent-reported outcome measures such as a validated parent satisfaction questionnaire and other parent-reported outcome measures.^124^ The final phase will then involve a Delphi Survey to determine a COS by consensus recommendation with input from all relevant stakeholders including parents and former patient.
